# Acute anxiety and autonomic arousal induced by CO_2_ inhalation impairs prefrontal executive functions in healthy humans

**DOI:** 10.1038/s41398-019-0634-z

**Published:** 2019-11-12

**Authors:** George Savulich, Frank H. Hezemans, Sophia van Ghesel Grothe, Jessica Dafflon, Norah Schulten, Annette B. Brühl, Barbara J. Sahakian, Trevor W. Robbins

**Affiliations:** 1 Department of Psychiatry, University of Cambridge, School of Clinical Medicine, Cambridge, UK; 2 Behavioural and Clinical Neuroscience Institute, University of Cambridge, Cambridge, UK; 3 MRC Cognition and Brain Sciences Unit, University of Cambridge, Cambridge, UK; 4 Department of Psychology, University of Cambridge, Cambridge, UK

**Keywords:** Neuroscience, Psychology

## Abstract

Acute anxiety impacts cognitive performance. Inhalation of air enriched with carbon dioxide (CO_2_) in healthy humans provides a novel experimental model of generalised anxiety, but has not previously been used to assess cognition. We used inhalation of 7.5% CO_2_ to induce acute anxiety and autonomic arousal in healthy volunteers during neuropsychological tasks of cognitive flexibility, emotional processing and spatial working memory in a single-blind, placebo-controlled, randomized, crossover, within-subjects study. In Experiment 1 (*n* *=* 44), participants made significantly more extra-dimensional shift errors on the Cambridge Neuropsychological Test Automated Battery (CANTAB) Intra-Extra Dimensional Set Shift task under CO_2_ inhalation compared with ‘normal’ air. Participants also had slower latencies when responding to positive words and made significantly more omission errors for negative words on the CANTAB Affective Go/No-go task. In Experiment 2 (*n* = 28), participants made significantly more total errors and had poorer heuristic search strategy on the CANTAB Spatial Working Memory task. In both experiments, CO_2_ inhalation significantly increased negative affect; state anxiety and fear; symptoms of panic; and systolic blood pressure/heart rate. Overall, CO_2_ inhalation produced robust anxiogenic effects and impaired fronto-executive functions of cognitive flexibility and working memory. Effects on emotional processing suggested a mood-congruent slowing in processing speed in the absence of a negative attentional bias. State-dependent effects of anxiety on cognitive-emotional interactions in the prefrontal cortex warrant further investigation.

## Introduction

Emotion and cognition are closely integrated phenomena, such that emotions influence, and are influenced by, cognitive processes^1–3^. Executive functions heavily rely on the frontal lobes and are necessary for optimal selection, organisation and monitoring of actions for attaining goals^4–6^. However, negative emotional states, such as anxiety, increase arousal and corresponding autonomic responses and can also bias cognitive processes in favour of selectively prioritising negative information^7,8^. Anxiety in particular enhances vigilance when detecting emotionally-salient information in the environment, but disrupts working memory^9,10^. Although anxiety can mediate adaptive behaviour in response to threat, it can also impair core aspects of cognition through its profound influence on prefrontal executive functions^11^.

Deficits in executive functions have been found in anxiety disorders^12–14^. Acute anxiety impairs cognitive flexibility, the ability to adapt one’s behaviour in response to rapid changes in the environment^15^. Individuals with high-trait anxiety favour recently acquired responses, even when they are no longer relevant^16^. Reduced cognitive flexibility has been proposed to be undermined by interference from irrelevant stimuli, in which anxiety prioritises stimulus-driven (bottom-up) attention over and above goal-directed (top-down) attention^16–18^. Emotionally-salient cues are known to bias attention, such that high-trait anxious individuals will selectively attend to negative information that matches and exacerbates their emotional state^19^. One measure of emotional processing is the Affective Go/No-go task^20,21^. Using this task, patients with depression have shown to make more omission errors when responding to happy than to sad words and respond more quickly to sad targets^22^. Patients with depression have also shown an inability to shift their attention from one affective valence to another, further supporting mood-congruent processing of negative stimuli^20^. In healthy volunteers, findings support an affective bias for positive information, as shown by faster responses for happy faces^23^.

Effects of acute anxiety on working memory have been mixed, with some studies showing anxiety-inducing impairments, e.g., see refs. ^24–27^. Discrepant findings are likely due to different paradigms being used for manipulating emotional states, including variations in delivery and length of delay between the acute stressor and cognitive assessment^28^. Negative affect has been hypothesized to selectively deplete processing resources required for adequate working memory performance^27,29,30^. Classic findings demonstrate that anxiety improves performance on simpler and well-rehearsed tasks, but impair performance on tasks that require complex, flexible thinking^31^. More generally, behavioural performance improves with low levels of arousal, but decreases with higher levels through deleterious effects on cognitive processes such as working memory.

One safe, reliable and robust method for inducing acute anxiety and autonomic arousal is through the inhalation of a gas mixture with an enhanced concentration of carbon dioxide^32,33^. Air enriched with CO_2_ has previously shown to evoke anxiety-related symptoms in healthy volunteers^34–36^ and in patients with anxiety disorders^37,38^. Acute administration of the benzodiazepine agonist lorazepam and chronic administration of the selective serotonin re-uptake inhibitor (SSRI) paroxetine has been shown to attenuate the effects of CO_2_ inhalation on state anxiety in healthy volunteers^39^, thus providing an experimental model of generalised anxiety^40^. However, the effects of CO_2_ inhalation on cognitive performance are less well characterised. Only one laboratory study has used an emotional antisaccade task to show that inhalation of 7.5% CO_2_ selectively increases attention to threat in healthy humans^41^.

The aim of our study was to characterise impairments in fronto-executive functions in healthy humans using an experimental manipulation analogous to generalised anxiety. We report two experiments investigating the effects of 7.5% CO_2_ inhalation on cognitive flexibility, emotional processing and spatial working memory in healthy human volunteers. Tasks were selected based on previous studies showing prefrontal and amygdalar dysfunction in highly anxious individuals and in patients with generalised anxiety disorder (GAD)^42,43^. We hypothesized that compared with a ‘normal air’ control condition; CO_2_ inhalation would impair cognitive flexibility and spatial working memory and induce mood-congruent processing of negative information. We further hypothesized, consistent with the literature^34,39–41^, that CO_2_ inhalation would increase negative affect; state anxiety and fear; symptoms of panic; and cardiovascular measures associated with somatic anxiety.

## Materials and methods

### Participants

Seventy-two healthy volunteers were recruited via mailing lists, posted flyers and from the Behavioural and Clinical Neuroscience Institute research database. Inclusion criteria were no current or past medical, psychiatric or neurological conditions or substance abuse. Exclusion criteria were pregnancy, currently smoking and having a first-degree relative diagnosed with a panic disorder. Participants were free of regular medication intake, but use of the oral contraceptive pill was accepted. These criteria were screened using the Mini-International Neuropsychiatric Inventory^44^; and by telephone interview. Invited participants were asked to abstain from alcohol consumption 36 h prior to the experiment as well as caffeinated drinks from the midnight before testing. All participants provided written informed consent.

### Gas mixture

Air enriched with CO_2_ (7.5% CO_2_, 21% O_2_, 71.5% N_2_) was used to evoke somatic anxiety^34^ and was stored in 10 L cylinders compressed with 200 bar. The air condition (control) consisted of approximately 0.0016% CO_2_, 21% O_2_ and 78% N_2_.

### Neuropsychological measures

We tested cognitive tasks in two cohorts of healthy participants. Participants performed parallel versions of cognitive tasks from the computerised Cambridge Neuropsychological Test Automated Battery (CANTAB; www.cambridgecogntion.com) in the two gas inhalation sessions. Tasks were performed over two experiments given the limited time window to safely assess cognitive performance during the CO_2_ challenge. Inhalation of CO_2_ for up to 20 min is the maximal inhalation duration known to be safely tolerated without serious side effects (e.g., see refs. ^34,37,^^39–41^).

### Experiment 1

#### CANTAB Intra-dimensional/Extra-dimensional Set-shifting task (IDED)

The CANTAB IDED task^45^ is a measure of rule acquisition and reversal. It features visual discrimination, attentional set formation, maintenance of attention, set shifting and flexibility. Two artificial dimensions are used: colour-filled shapes and white lines. Simple stimuli are made of just one of these dimensions, whereas compound stimuli are made of both, namely white lines overlying colour-filled shapes. Participants must use feedback to work out a rule that determines which stimulus is correct. After six correct responses, the stimuli and/or rule changes. Initially the task will involve simple stimuli which are made up of just one of the dimensions. Later on, compound stimuli are used. The shifts in rule are firstly ‘intra-dimensional’ and then secondly ‘extra-dimensional.’ Outcome measures include the total errors made; errors made in the critical stages of intra-dimensional set shift; errors made in the critical stages of extra-dimensional set shift; and the number of errors made prior to the extra-dimensional shift of the task.

#### CANTAB Affective Go/No-go task (AGN)

The CANTAB AGN task^20^ is a measure of information-processing biases for positive and negative stimuli. The task consists of several blocks, each of which presents a series of words from two of three different affective categories: positive (e.g. joyful), negative (e.g. burden) or neutral (e.g. pause). The participant is given a target category and is asked to select a word when it matches this category. Outcome measures include errors of commission (an incorrect response to a distractor stimulus on ‘No/go’ trials) and omission (no response to a target stimulus on ‘Go’ trials) and latency (speed of response).

### Experiment 2

#### CANTAB Spatial Working Memory task (SWM)

The CANTAB SWM^46^ is a measure of ability to retain spatial information and to manipulate remembered items in working memory. It is a self-ordered task, which also assesses heuristic strategy. A number of coloured boxes are first shown on the screen. Participants are instructed to find a blue token in each box, using a process of elimination, and to use them to fill up an empty column on the right side of the screen. The colour and position of the boxes are changed from trial to trial (with the number of boxes increasing). Outcome measures include total errors (selecting boxes that have already been found to be empty and revisiting boxes which have already been found to contain a token) and strategy (a predetermined sequence by beginning with a specific box and then, once a blue token has been found, to return to that box to start the new search sequence).

### Questionnaire measures

Administered trait measures included Spielberger’s Trait-Anxiety Inventory [STAI-T; ref. ^47^], Beck’s Depression Inventory [BDI; ref. ^48^] and the Intolerance of Uncertainty Scale [IUS; ref. ^49^]. The STAI-T is a 20-item self-report measure of trait anxiety, with higher scores indicating higher anxiety levels (range 20–80); the BDI is a 21-item self-report measure of depression, with higher scores indicating higher levels of depression severity (range 0–63); and the IUS is a 12-item self-report measure of responses to uncertainty, ambiguous situations and the future (e.g. ‘When it’s time to act, uncertainty paralyses me’), with higher scores indicating less tolerance (range 12–60). State measures included the Negative Affect subscale of the Positive and Negative Affect Schedule [PANAS; ref. ^50^; a 10-item measure of current negative affect, with items such as irritability, distress and nervousness enabling a more comprehensive measure of negative emotionality induced by CO_2_ inhalation^41^; range 10–50]; the Acute Panic Inventory [API; ref. ^51^; a 17-item measure of the severity of symptoms that typically occur during spontaneous panic attacks, with scores ranging from 0 = symptom not experienced to 3 = severe experience of symptom; e.g. ‘Do you have rapid or difficulty breathing’; range 0–51]; and 10 cm visual analogue scales of state anxiety, fear and happiness (higher scores indicate a greater emotional response).

### Procedure

This study received full ethical approval from the University of Cambridge Psychology Research Ethics committee (Pre.2013.98). This study was a single-blind, placebo-controlled, randomised, within-subject crossover design. All participants provided written informed consent.

For the gas sessions, the experimenter installed the mask used for inhaling the mixture on the participant’s face. Participants were asked to breathe through a soft silicon rubber nasal-oral mask, which was attached to a tube that led to a 100 L Douglas bag. Participants faced a screen, with the gas bag positioned behind it. In the control condition, participants breathed room air and pre-recorded sounds of gas being released from the cylinder were played in the background. In the CO_2_ condition, the valve of the gas bag was switched, such that the participant breathed the gas mixture from the Douglas bag. A cylinder with a compressed gas mixture was used to keep the bag filled. For safety reasons, the participant was accompanied by at least two researchers and a carbon dioxide safety monitor was used to monitor the CO_2_ concentration in the testing room throughout the entire experiment. Systolic blood pressure (mmHG) and heart rate (beats per minute) were measured using a digital blood pressure monitor.

Baseline trait (anxiety, depression, intolerance of uncertainty), panic (API) and cardiovascular (heart rate, blood pressure) measures were first collected in this fixed order pre-inhalation (5 min). Each gas session then comprised cognitive testing, state (panic, affect, mood) and cardiovascular (as above) measures, which lasted a maximum duration of 20 min. A 5–10-min break was given in between the inhalation sessions. Participants in Experiment 1 completed the CANTAB IDED and AGN tasks and participants in Experiment 2 completed the CANTAB SWM test. Gas administration was counterbalanced separately by gender using two orders (CO_2_/air, air/CO_2_) randomly generated across experiments. Participants were given a 5–10-min rest period between the inhalation sessions. After the two inhalation sessions, participants were debriefed about their gas administration order. All participants were paid £8/h and thanked for their time.

### Statistical analyses

With alpha set at 0.05 and 80% power, an a priori power analysis based on a previous study^41^ found that 15 participants would be sufficient to detect a within-subjects effect of CO_2_ inhalation on negative affect (*η*_p_^2^ = 0.31). Paired samples *t*-tests were used to investigate within-subject differences in cognitive performance under CO_2_ and ‘normal’ air inhalation. To test the effect of the gas manipulation on negative affect, panic symptoms and autonomic arousal, repeated-measures ANOVAs were used with ‘Time’ as the within-subjects factor with three levels (baseline/pre-inhalation, CO_2_, and air). Main effects were further compared between mean scores under CO_2_ and mean scores at baseline and under air separately, adjusting confidence intervals using Bonferroni correction for multiple comparisons. Paired samples *t*-tests were performed to investigate state anxiety, fear and happiness under CO_2_ and air. Correlational and regression analyses were used to examine potential associations between cognitive performance and baseline trait measures, the degree of anxiety/panic symptoms reported and cardiovascular effects experienced under CO_2_. Due to the high number of neuropsychological test outcome measures, the Benjamini-Hochberg procedure^52^ was applied at *q* < 0.05 to control for false discovery; significant *p*-values remained (all two-sided).

## Results

### Participant characteristics

Experiment 1 was composed of 22 males and 22 females with a mean age of 29.25 years (SD = 10.66) and Experiment 2 was composed of 13 males and 14 females with a mean age of 26.78 years (SD = 9.94). All participants had completed at least pre-University education at the time of testing with an average of 18.25 years in education (SD = 1.26) in Experiment 1 and 17.17 years in education (SD = 2.57) in Experiment 2. As expected, mean trait-anxiety and depression scores were within normal ranges for healthy adults (STAI-T: Experiment 1 = 34.98, SD = 6.24; Experiment 2 = 31.86, SD = 7.70; normative score = 33.0, SD = 9.4; ref. ^53^; and BDI: Experiment 1 = 4.63, SD = 4.75; Experiment 2 = 2.68, SD = 3.08; minimal range = 0–13). Furthermore, mean intolerance of uncertainty scores (Intolerance of Uncertainty Scale) were 27.51 (SD = 8.27) in Experiment 1 and 26.32 (SD = 7.38) in Experiment 2. Age, gender, years in education, trait-anxiety, depression and intolerance of uncertainty were not significantly different between experiments (all *p*’s > 0.06). In both samples, baseline (pre-inhalation) measures of trait- and state anxiety, negative affect and panic symptoms were not significantly different between the two gas administration orders (all *p*’s > 0.09). Furthermore, gas order did not interact with the effects of CO_2_ on measures of state anxiety and fear, negative affect and panic (all *p*’s between 0.07 and 0.75) in either experiment.

### Negative affect and mood state

**Fig. 1:**
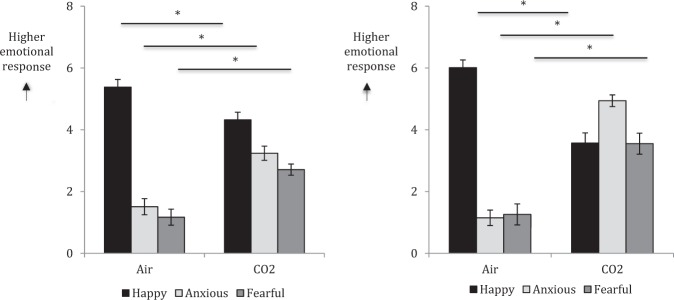
Visual analogue scales of state happiness, anxiety and fear during air and CO_2_ inhalation in Experiment 1 (left) and Experiment 2 (right); in both experiments, state anxiety and fear were significantly increased under CO_2_, and state happiness was significantly decreased

Means and standard deviations for negative affect at each time point are presented in Table 1. In Experiment 1, there was a main effect of Time, *F*(2, 40) = 9.40, *p* < 0.001, *η*_p_^2^ = 0.32, such that negative affect was significantly higher under CO_2_ compared with baseline, *p* < 0.001 (mean difference = 2.81) and air, *p* = 0.003 (mean difference = 2.64). Similarly in Experiment 2, there was a main effect of Time, *F*(2, 26) = 17.79, *p* < 0.001, *η*_p_^2^ = 0.56, with negative affect again being significantly higher under CO_2_ compared with baseline, *p* = 0.005 (mean difference = 5.34) and air, *p* < 0.001 (mean difference = 7.04). In both experiments, state anxiety and fear were significantly increased under CO_2_ compared with air (all *p*’s < 0.001), whereas state happiness was significantly decreased under CO_2_ compared with air (all *p*’s < 0.001) (Fig. 1). In both experiments, women gave significantly higher ratings for state fear than men (*p* = 0.03 and 0.007, respectively) during CO_2_ inhalation.

**Table 1:** Means and standard deviations for measures of negative affect, panic symptoms, blood pressure and heart rate by experiment

	Experiment 1	Experiment 2
Negative affect (NA)		
Baseline	12.00 (2.32)	12.89 (2.36)
Air	12.20 (3.91)	11.39 (2.10)
CO_2_	14.84 (4.87)*	18.43 (8.06)*
Panic symptoms (API)		
Baseline	1.60 (2.80)	2.29 (2.99)
Air	2.98 (3.67)	2.86 (3.95)
CO_2_	10.18 (7.34)*	17.54 (8.32)*
Systolic blood pressure		
Baseline	116.72 (12.86)	119.22 (13.12)
Air	114.07 (13.75)	116.46 (11.27)
CO_2_	128.30 (17.91)*	140.82 (21.54)*
Heart rate		
Baseline	70.45 (10.63)	71.93 (10.29)
Air	74.28 (11.72)	73.25 (9.42)
CO_2_	76.98 (12.59)**	91.89 (17.61)*

*NA* Negative Affect Schedule subscale, *API* Acute Panic Inventory

* denotes significant difference between CO_2_ and baseline only; ** denotes significant difference between CO_2_ relative to baseline and air

### Panic symptoms and autonomic arousal

Means and standard deviations for panic and arousal measures at each time point are presented in Table 1. In Experiment 1, there was a main effect of Time for the API, *F*(2, 40) = 41.35, *p* < 0.001, *η*_p_^2^ = 0.68. As expected, mean panic symptoms were significantly higher under CO_2_ compared with baseline, *p* < 0.001 (mean difference = 8.57) and air, *p* < 0.001 (mean difference = 7.17). CO_2_ inhalation also increased cardiovascular measures of arousal. There were main effects of Time for both systolic blood pressure, *F*(2, 41) = 34.52, *p* < 0.001, *η*_p_^2^ = 0.63 and heart rate, *F*(2, 41) = 10.47, *p* < 0.001, *η*_p_^2^ = 0.34. Mean systolic blood pressure was significantly higher under CO_2_ compared with baseline, *p* < 0.001 (mean difference = 11.58) and air, *p* < 0.001 (mean difference = 14.23). However, mean heart rate under CO_2_ only significantly differed from mean heart rate at baseline, *p* < 0.001 (mean difference = 6.51).

In Experiment 2, there was a main effect of Time for the API, *F*(2, 26) = 50.67, *p* < 0.001, *η*_p_^2^ = 0.80. Mean panic symptoms were significantly higher under CO_2_ compared with baseline, *p* < 0.001 (mean difference = 15.25) and air, *p* < 0.001 (mean difference = 14.68). Again, there were main effects of Time for systolic blood pressure, *F*(2, 25) = 27.08, *p* < 0.001, *η*_p_^2^ = 0.68 and heart rate, *F*(2, 25) = 18.07, *p* < 0.001, *η*_p_^2^ = 0.59. Mean systolic blood pressure was significantly higher under CO_2_ compared with baseline, *p* < 0.001 (mean difference = 21.48) and air, *p* < 0.001 (mean difference = 24.15). Mean heart rate was also significantly higher under CO_2_ compared with baseline, *p* < 0.001 (mean difference = 19.52) and air, *p* < 0.001 (mean difference = 18.04).

### Cognitive measures

#### Experiment 1

##### Cognitive flexibility (CANTAB IDED)

**Fig. 2:**
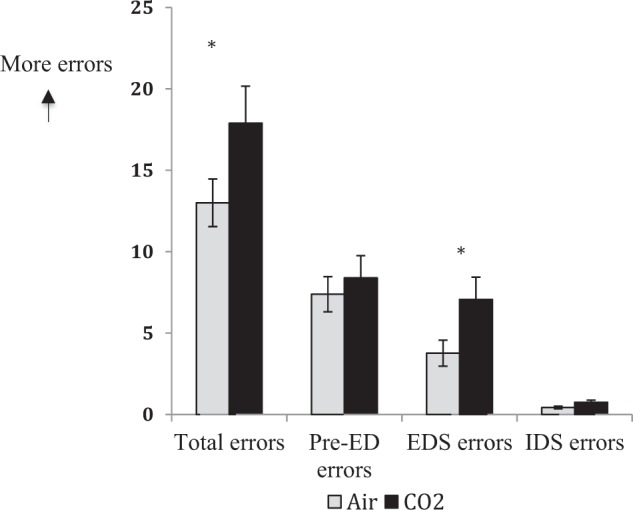
Participants made significantly more total errors and errors in the extra-dimensional stage of the CANTAB IDED task under CO_2_ compared with air; the number of errors made prior to the extra-dimensional stage and intra-dimensional shift errors were not significantly different (Experiment 1)

A paired samples *t*-test revealed that participants (Fig. 2) made significantly more total errors under CO_2_ compared with air, *t*(43) = 2.69, *p* = 0.01, *d* = 0.41 (CO_2_ mean = 17.89, SD = 15.09; air mean = 13.00, SD = 9.70). Participants also made significantly more extra-dimensional shift errors under CO_2_ compared with air, *t*(43) = 2.63, *p* = 0.01, *d* = 0.40 (CO_2_ mean = 7.07, SD = 9.08; air mean = 3.77, SD = 5.30). Pre-ED errors were not significantly different, *t*(43) = 0.62, *p* = 0.54 (CO_2_ mean = 8.39, SD = 9.08; air mean = 7.39, SD = 7.14). Furthermore, the mean number of intra-dimensional shift errors under CO_2_ and air was not significantly different, *t*(43) = 1.86, *p* = 0.07 (CO_2_ mean = 0.75, SD = 0.84; air mean = 0.43, SD = 0.55.

##### Affective bias (CANTAB AGN)

**Fig. 3:**
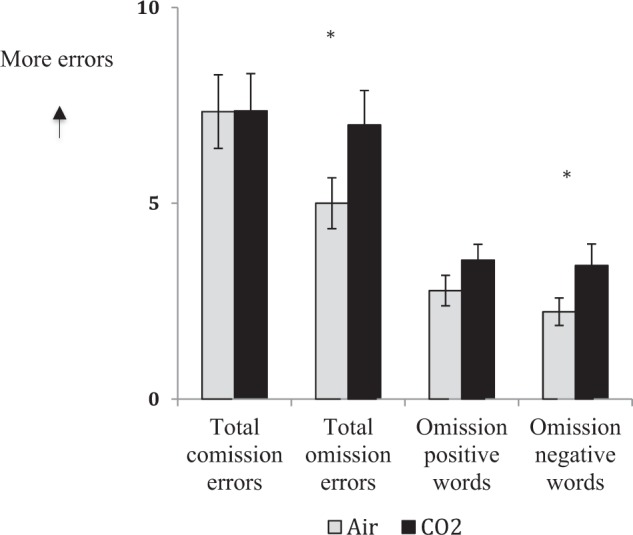
Participants made more total omission errors and omission errors for negative words on the CANTAB AGN task under under CO_2_ compared with air; total commission errors and omission errors for positive words were not significantly different (Experiment 1)

Commission errors were not significantly (Fig. 3) different, *t*(43) = 0.03, *p* = 0.98 (CO_2_ mean = 7.34, SD = 6.27; air mean = 7.34, SD = 6.21). However, participants made significantly more omission errors under CO_2_ compared with air, *t*(43) = 3.31, *p* = 0.002, *d* = 0.50 (mean CO_2_ = 6.95, SD = 5.85; mean air = 5.00, SD = 4.29). Follow-up paired samples *t*-tests revealed significantly more omission errors for negative words under CO_2_ compared with air, *t*(43) = 2.96, *p* = 0.005, *d* = 0.45 (mean CO_2_ = 3.41, SD = 3.65; mean air = 2.23, SD = 2.34), with mean omission errors for positive words not reaching significance, *t*(43) = 1.87, *p* = 0.07 (mean CO_2_ = 3.55, SD = 3.10; mean air = 2.77, SD = 2.55. Mean latencies for correct responses were significantly slower for positive words under CO_2_ compared with air, *t*(43) = 2.67, *p* = 0.01, *d* = 0.04 (mean CO_2_ = 591.65, SD = 65.33; mean air = 573.16, SD = 67.64), but not negative words, *t*(43) = 1.65, *p* = 0.11 (mean CO_2_ = 600.29, SD = 76.80; mean air = 586.80, SD = 66.45).

#### Experiment 2

##### Spatial working memory (CANTAB SWM)

**Fig. 4:**
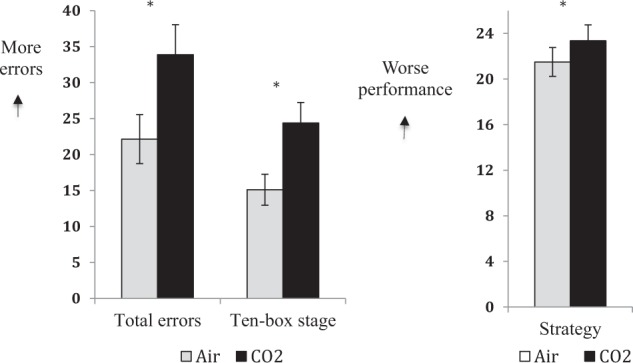
Participants made significantly more total errors and errors at the highest level of difficulty (ten-box stage) on the CANTAB SWM task under CO_2_ compared with air (left); participants also showed poorer search strategy under CO_2_ compared with air (right) (Experiment 2)

Participants made significantly more total (Fig. 4) errors under CO_2_ compared with air, *t*(27) = 3.40, *p* = 0.002, *d* = 0.63 (CO_2_ mean = 33.89, SD = 21.97; air mean = 22.14, SD = 18.01). Follow-up within-subject comparisons at each level of difficulty revealed that participants made significantly more errors at the hardest task level (ten-box stage) under CO_2_ compared with air, *t*(27) = 3.19, *p* = 0.004, *d* = 0.60 (CO_2_ mean = 24.39, SD = 15.06; air mean = 15.11, SD = 11.45). Within-subject performance at lower levels of difficulty (eight-, six-, and four-box stages) was not significantly different (all *p*’s > 0.16). Participants showed an inferior heuristic search strategy under CO_2_ compared with air, *t*(27) = 2.38, *p* = 0.03, *d* = 0.45 (CO_2_ mean = 23.36, SD = 7.39; air mean = 21.50, SD = 6.73).

##### Whole sample analyses (performed separately by task)

Correlational analyses revealed that outcome measures from the IDED and SWM tasks were not significantly associated with symptoms of panic, state anxiety/fear or cardiovascular measures during CO_2_ inhalation (all *p*’s ≥ 0.05). However, on the AGN task, significant associations were found between total commission errors and negative affect (*r* = 0.47, *p* = 0.001), panic symptoms (*r* = 0.37, *p* = 0.01) and state fear (*r* = 0.32, *p* = 0.04), and total omission errors and negative affect (*r* = 0.41, *p* = 0.006) during CO_2_ inhalation. A regression analysis revealed that baseline intolerance of uncertainty significantly predicted omission errors for negative words under CO_2_, *β* = 0.32, *t* = 2.15, *p* *=* 0.04 (the model accounted for 32% of the variance and was significant, *F*(1, 42) = 4.62, *p* = 0.04).

Lastly, post hoc power analyses revealed that Experiment 1 achieved 74% power and Experiment 2 achieved 86% power based on key within-group effects of CO_2_ on IDED extra-dimensional shift errors and SWM errors made at the ten-box stage, respectively.

## Discussion

We investigated the effects of experimentally induced acute anxiety and autonomic arousal on core fronto-executive functions in healthy humans as a model of those underlying generalised anxiety. As hypothesized, we found that compared with baseline and ‘normal’ air, inhalation of air enriched with 7.5% CO_2_ significantly increased negative affect, state anxiety/fear, symptoms of panic, and cardiovascular measures of systolic blood pressure and heart rate. Conversely and as expected, CO_2_ inhalation also significantly reduced state happiness. On the CANTAB IDED task, CO_2_ inhalation significantly increased the number of extra-dimensional, but not intra-dimensional, shift errors. Participants also made significantly more total errors and had an inferior heuristic search strategy on the CANTAB SWM task. Evidence of a mood-congruent processing bias was mixed: on the CANTAB AGN task, we found that participants responded more slowly to positive words, but made more omission errors for negative words. Correlational analyses revealed significant associations between impairments on the AGN task and negative affect, panic symptoms and state fear under CO_2_, suggesting that cognitive effects were influenced by emotional rather than cardiovascular changes. Throughout both experiments, the impact of CO_2_ was highly present during its inhalation, but disappeared immediately upon cessation, thereby demonstrating that anxiety induction was acute rather than chronic and with good reproducibility and safety.

Studies to date have shown evidence of anxiety-induced impairments on cognitive flexibility^54–56^. Participants in our study generally made very few, if any, intra-dimensional shift errors, indicating rather specific effects on attentional set shifting rather than discrimination learning per se^57^. Impaired extra-dimensional shifting is characteristic of adult patients with obsessive-compulsive disorder (OCD) and their unaffected first-degree relatives, suggesting that cognitive flexibility is a candidate endophenotype that exists even in the absence of clinically significant symptoms^58^. Our data raise the possibility that cognitive flexibility may also be impaired in generalised anxiety disorder (GAD), which might reflect specific dysfunction in prefrontal cortical regions. Indeed, poor attentional flexibility for extra-dimensional shifting is sensitive to frontal lobal injury^59^ and related to distinctly weakened functional connectivity between the caudate and the ventrolateral prefrontal cortex^60^. The effects of CO_2_ inhalation on emotional processing revealed slower responding to positive words, but significantly more omission errors for negative words. However, both total omission and commission errors were highly associated with negative emotions and panic under CO_2_ across the samples of each experiment separately. Healthy individuals are characterised by a positive attentional bias and have previously been shown to make more omission errors in response to sad stimuli than to happy stimuli, whereas patients with depression show the reverse pattern of responding^20,22^. Our data provide some evidence of an affective bias *congruent with* negative mood induction, such that healthy participants showed a slowing of response to positive words under CO_2_. Although CO_2_ inhalation is an emotional manipulation, an overall adaptive ‘healthy’ attentional bias may confer resilience against negative emotional processing. This may in part explain why less intolerance of uncertainty, a key construct impaired in GAD, OCD, panic and other emotional disorders, significantly predicted omission errors for negative words only (e.g. rejection of uncertainty/ambiguity protects against the processing of negative emotional information).

Theoretical and neurophysiological perspectives suggest that anxiety impairs working memory by competing for processing resources via modulation of prefrontal cortical network functioning^29^. As expected, we found that participants committed significantly more total errors in our spatial working memory task during CO_2_ inhalation compared with ‘normal’ air inhalation. Furthermore, this effect was driven by a significant difference at the hardest level of task difficulty (i.e. the ten-box stage). Participants under CO_2_ inhalation also exhibited a significantly worse ‘strategy’ score, meaning they more frequently started a new trial by searching for a token in a new box rather than following a more systematic search strategy^46^. Others have shown that increased cortisol or adrenergic activity impairs working memory^61^ and that alpha-1 receptor agonists can impair the spatial delayed response task in rhesus monkeys^62^. In general, alpha-1 activity tend to promote relatively automatic conditioned avoidance behaviours and habitual or ritualistic behaviours^62,63^.

Our data have important clinical implications. They generally contribute to the existing literature on the interaction between anxiety and cognitive processes using an experimental model that readily translates between animals and humans. More specifically, CO_2_ inhalation safely and quickly induces a maladaptive level of anxiety and arousal that impacts executive functioning similar to psychiatric disorders. As such, this model can be used to translate the efficacy of novel compounds from preclinical models to healthy human volunteers prior to clinical trials in patients (e.g. evaluation of new anxiolytic treatments such as beta-adrenergic agonists for anxiety disorders^35^). However, it should be noted that mood and panic symptoms in the present sample were below the values typically seen in clinical populations, making it difficult to generalise to clinical levels of anxiety. Others have suggested that higher concentrations of CO_2_ may provide a better model of panic disorder (e.g. 35%)^38,64^, although similar effects on panic-like symptoms have been observed using 7.5%^34,37^. Future work could investigate the potential differential effects of anxiety types (e.g. somatic vs. psychic; central vs. peripheral) on cognitive performance during CO_2_ inhalation as well as the neural circuitry implicated in the underlying mechanisms of anxiety when performing complex executive tasks (e.g. dorsolateral prefrontal cortex and lateral parietal cortex)^65^. Other research outside of the scope of the present study could disentangle psychological/emotional effects, interference from physical sensations and physiological changes in respiratory or autonomic function when interpreting cognitive changes. With respect to emotional processing, it has been previously shown that threat is associated with over-activation of the amygdala in highly anxious individuals^41^. Although our data showed that ratings of anxiety and fear significantly increased during CO_2_ inhalation (with ratings of happiness significantly decreasing), it is important to note that the stimuli used in the present study were generally negative rather than threatening. Achieving selective attentional effects may therefore require amygdala reactivity following anticipation or provocation of threat not induced by CO_2_ inhalation.

Limitations include single-blind administration of the gas manipulation (for safety reasons) and unequal sample sizes between experiments (although both achieved adequate power). Prior to debriefing, most participants reported that the CO_2_ inhalation session was a relatively intense and/or unpleasant experience, which may have increased demand characteristics of the air inhalation session, although sessions were counterbalanced to reduce this and gas administration order did not interact with key measures of subjective anxiety and panic. Finally, our sample mostly comprised young adults, which may not represent the wider population, but does capture a group in which anxiety disorders are increasingly prevalant. Although a gender difference was only found on subjective ratings of state fear during CO_2_ inhalation, more detailed investigation of their effects on cognitive/emotional responses to acute anxiety are warranted in larger samples.

Overall, the present study demonstrated state-dependent effects of acute anxiety and autonomic arousal on fronto-executive functions in healthy humans, including impaired cognitive flexibility and working memory. Effects on emotional processing showed a mood-congruent slowing of response in the absence of a negative attentional bias. Identification of resilience factors that protect against acute anxiety may help promote cognitive performance needed for achieving optimal behavioural performance. 7.5% CO_2_ inhalation in healthy humans also provides a robust model of generalised anxiety that could be used to test new drug therapies for its treatment.
